# *Streptococcus agalactiae*

**DOI:** 10.1155/S1064744993000146

**Published:** 1993

**Authors:** Sebastian Faro


Infectious Diseases in Obstetrics and Gynecology 1:59 (1993)
© 1993 Wiley-Liss, Inc.

### *Streptococcus agalactiae*

*Streptococcus agalactiae,* a unique bacterium commonly referred to as group B streptococcus (GBS), is evolving to become a major pathogen that causes a wide variety of problems across a broad spectrum of individuals.

The greatest controversy over this bacterium involves the obstetrical patient. Lawyers have made this organism a centerpiece, leading their clients to believe that the presence of GBS should always be sought and detected, thereby allowing the physician the opportunity to prevent the diseases it causes. Their position has been supported by our pediatric colleagues, who recommend universal screening of all pregnant patients. These proponents of universal screening appear not to understand that inaccuracies surround the detection of this bacterium or that the infant may be infected prior to birth. The fact remains that universal screening cannot be implemented until an inexpensive but highly accurate method has been developed to accomplish such screening.

At present, the gold standard is the use of enrichment techniques in obtaining cultures of the organism from the lower genital tract. However, no agreement has been reached as to when in pregnancy the patient should be screened. This dilemma is due both to our inability to detect extremely low numbers of colonizing bacteria and to the transitional state that characterizes some colonized patients. Rapid tests have proved to be unreliable because they are associated with a significant false-negative rate. False-positive tests are also of concern because antibiotics would be administered to individuals not needing therapy, placing them at risk for adverse reactions, some of which may be serious and even life-threatening.

The recommendation of administering antibiotic therapy to all positively colonized laboring patients has evolved from the earlier recommendation of treating high-risk patients. In the meantime, legal pressure has created a debate on whether or not patients should be screened and all colonized patients should be treated. At the same time, large randomized, blinded studies on these specific issues are lacking.

There is no doubt that this is an emotional issue from the patient's point of view, nor is there any doubt that this bacterium should be prevented from causing infection not only in the newborn and mother, but also in the gynecologic patient scheduled for pelvic surgery. Not only has the legal profession not helped to find a scientific solution, it has caused many physicians to undertake management programs that are not beneficial or safe or are not effective or cost-effective. Although we agree that the patient at risk should be managed aggressively, recommendations for mass screening should be held in abeyance until more specific, sensitive, and economical tests have been developed and proved to be reliable.

*Sebastian Faro*
*Editor-in-Chief*

